# Bias-corrected monthly precipitation data over South Siberia for 1979-2019

**DOI:** 10.1016/j.dib.2021.107440

**Published:** 2021-10-05

**Authors:** Anna Ryazanova, Nadezhda Voropay, Egor Dyukarev

**Affiliations:** a Institute of Monitoring of Climatic and Ecological System SB RAS, Akademicheskii 10/3, Tomsk 634055, Russia; b V B Sochava Institute of Geography SB RAS, Ulan-Batorskaya st., 1, Irkutsk 664033, Russia; c Yugra State University, Chekhov st. 16, Khanty-Mansiysk 628012, Russia

**Keywords:** Precipitations, Reanalysis, South Siberia, Bias correction, Downscaling

## Abstract

Bias-Corrected Precipitation data over **S**outh **S**iberia (**CPSS**) contains monthly precipitation data for the area within the coordinates 50–65 N, 60–120 E for the period from January 1979 to December 2019. CPSS data were combined from monthly total precipitation data from ERA5 reanalysis European Centre for Medium-Range Weather Forecasts and precipitation data records from ground weather stations. The ERA5 data were scaled according to the derived scale coefficient. The linear scaling coefficient for each month and weather station were calculated and extrapolated to the study area using the ordinary kriging method. Data spatial resolution is 0.25° in the latitude and 0.25° in the longitude. CPSS reproduces the spatial variability of precipitation more precisely than can be done from the weather station observation network. The CPSS dataset will be useful for the study of extreme precipitation events and allow for more accurate hydrologic risk assessment at a regional level based on climate model results.

## Specifications Table

**Table:** 

Subject	Environmental Science: Climatology
Specific subject area	Precipitation, Gridded data, Hydrology, Water science
Type of data	Binary data (zipped NetCDF file)
How data were acquired	Data were combined from monthly total precipitation data from ERA5 reanalysis European Centre for Medium-Range Weather Forecasts (Copernicus Climate Change Service (C3S), 2017) and precipitation data records from ground weather stations (Il'yin et al., 2013). The ERA5 data were corrected according to the derived scale coefficient. The linear scaling coefficient for each month and weather station were calculated and extrapolated to the study area using the ordinary kriging method. For further details please see the related research article shown below (Voropay et al.,2021).
Data format	Raw
Parameters for data collection	Raw ERA5 ECMWF reanalysis data were bias corrected using data from ground weather stations network.
Description of data collection	Bias corrected gridded monthly precipitation data for the area within the coordinates 50–65 N, 60–120 E for the period from January 1979 to December 2019. Data spatial resolution is 0.25° × 0.25°.
Data source location	Country: Russia Latitude: from 50 N to 65 N Longitude: from 60 E to 120 E Primary data sources: Copernicus Climate Change Service (C3S), (2017) ERA5: Fifth generation of ECMWF atmospheric reanalyses of the global climate. Copernicus Climate Change Service Climate Data Store (CDS), Available at: https://cds.climate.copernicus.eu/cdsapp#!/home B.M.Il'yin, O.N.Bulygina, E.G. Bogdanova, V.M. Veselov, S.Y. Gavrilova, (2013) Dataset of monthly precipitation totals, with the elimination of systematic errors of precipitation gauges. Available at: http://meteo.ru/data/506-mesyachnye-summy-osadkov-s-ustraneniem-sistematicheskikh-pogreshnostej-osadkomernykh-priborov
Data accessibility	Repository name: Zenodo Data identification number: 10.5281/zenodo.4472613 Direct URL to data: https://zenodo.org/record/4472614
Related research article	N.N. Voropay, A.A. Ryazanova, E.A. Dyukarev, High-resolution bias corrected precipitation data over the South Siberia, Russia. Atmospheric Research 254, 105528. https://doi.org/10.1016/j.atmosres.2021.105528.

## Value of the Data

• The accuracy of global hydrometeorological data is important for regional and global climate studies.
• CPSS data were combined from monthly total precipitation data from ERA5 reanalysis and precipitation data records from ground weather stations.
• CPSS reproduces the spatial variability of precipitation more precisely than can be done from the weather station observation network.
• The CPSS dataset will be useful for the study of extreme precipitation events and allow for more accurate hydrologic risk assessment at a regional level based on climate model results.

## 1 Data Description

Dataset contains monthly precipitation data for the area within the coordinates 50–65 N, 60–120 E for the period from January 1979 to December 2019. Data spatial resolution is 0.25° in the latitude and 0.25° in the longitude. The data presented and described in this article is available for download from Zenodo https://zenodo.org/record/4472614 [4]. All data files are available in Network Common Data Form (NetCDF) format. Data files compressed into one zip archive.

File **CRSS_1979-2019.nc** contains the following variables:

Lat (float) [1 × 61] – latitude of grid points from 50 N to 65 N with step 0.25;
Lon (float) [1 × 241] – longitude of grid points from 60 E to 120 E with step 0.25;
Mon (float) [1 × 12] – number of months from January to December;
Y (float) [1 × 41] – years from 1979 to 2019;
MonTPrecCor (double) [41 × 12 × 61 × 241] – gridded total monthly precipitation for each month for year from 1979 to 2019.

File **CRSS_1979-2019_averaged.nc** contains the following variables:

Lat (float) [1 × 61] – latitude of grid points from 50 N to 65 N with step 0.25;
Lon (float) [1 × 241] – longitude of grid points from 60 E to 120 E with step 0.25;
Mon (float) [1 × 12] – number of months from January to December;
MeanMonTPrecCor (double) [12 × 61 × 241] – gridded total monthly precipitation for each month averaged for period from 1979 to 2019.

The variable type is given in the round bracket, and variable size is in square brackets. The precipitation amount is given in mm of water accumulated during a month.

**Fig. 1:**
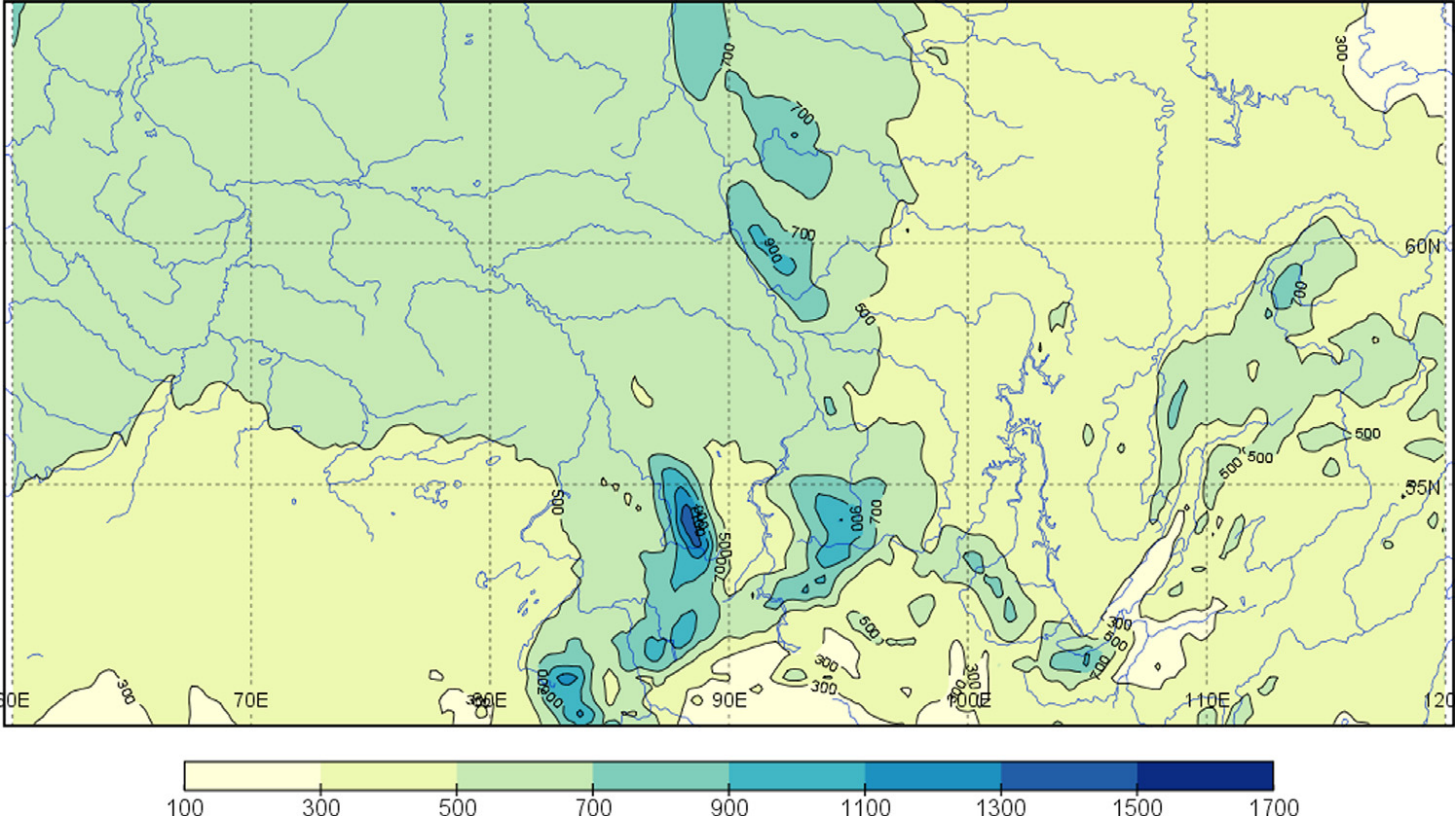
Map of annual precipitation (mm) over South Siberia for 1979–2019 according to CPSS.

**Fig. 2:**
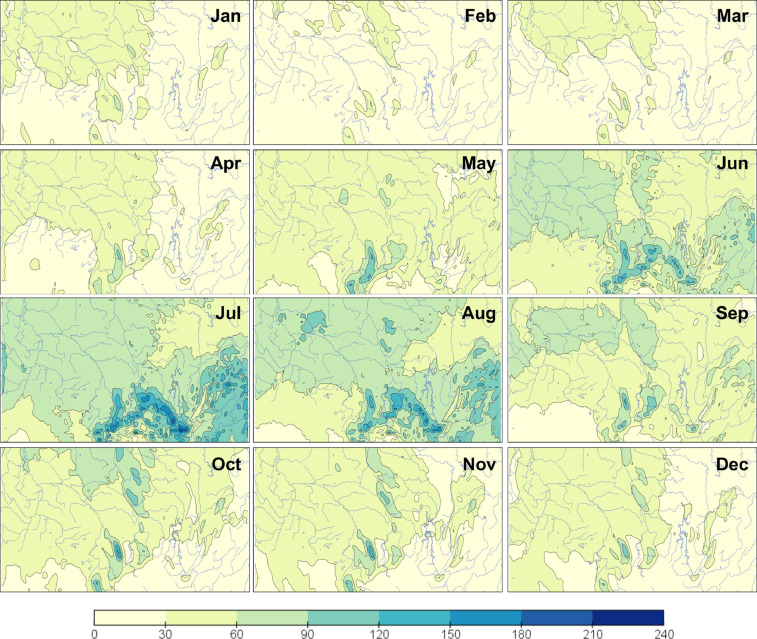
Monthly maps of precipitation (mm) over South Siberia for 1979–2019 according to CPSS.

The spatial distribution of a 45-year long-term sum of average of annual precipitation according to the corrected reanalysis CPSS is presented in Fig. 1, which shows the map over South Siberia for 1979–2019. Monthly precipitation maps are shown at Fig. 2.

## 2 Experimental Design, Materials and Methods

The modern weather forecasting systems produce large globally gridded datasets containing information about the conditions of the land and the atmosphere. These datasets have high spatial, temporal resolution, and continuous records. For example, gridded precipitation products are available from various national climatic centers over the world. The rain gauge observations at ground weather stations are the most direct and the most accurate source of information about the state of the climate. The evaluation of the performance of precipitation gridded data at a regional scale comparing with valid ground precipitation data is important for proper analysis of ongoing climatic changes. We compare reanalysis monthly precipitation data against corrected weather station observation data and suggest a spatiotemporally distributed linear scaling correction for the reanalysis data resulting in the creation of a new Bias-Corrected Precipitation database over South Siberia (CPSS).

The ERA5 reanalysis provides detailed information on meteorological characteristics with spatial resolution of 0.25° × 0.25°. Direct comparison of the ERA5 reanalysis monthly precipitation data [1] against observed precipitation data records from ground weather stations over South Siberia showed that the linear correlation coefficients between reanalysis and weather station data are high but mean values are biased [3]. The mean absolute error varies from −23 to 90 mm. In order to reduce biases between the ERA5 and observed monthly precipitation we suggest a correction procedure composed from the following steps:

1. Creation of ERA5 data subset.
2. Creation of ground observation data subset.
3. Interpolation of reanalysis regular grid data into weather station locations.
4. Calculation of bias correction parameter for weather stations.
5. Interpolation of bias correction parameter into regular grid.
6. Applying bias correction coefficient to ERA5 data subset.
7. Validation of correction results.

*1. Creation of ERA5 data subset*

The ERA5 reanalysis [1] provides detailed fields reasonably reproducing features of mesoscale precipitation structures related to topography and synoptic-scale patterns. ERA5 total precipitation is the sum of convective and large-scale precipitation. The time interval was from January 1979 to December 2019. The study area located within the coordinates 50–65 N, 60–120 E and represents the Southern part of Siberia – a part of the Asian territory of Russia. The ERA5 monthly total precipitation dataset with a spatial resolution of 0.25° × 0.25° for the study area and time interval was downloaded from the ECMWF [1] in Network Common Data Form (NetCDF) format.

*2. Creation of ground observation data subset*

Monthly data on observations of precipitation were obtained from the archive of the All-Russian Research Institute of Hydrometeorological Information – World Data Center (RIHMI–WDC) (http://www.meteo.ru) [2]. Dataset of monthly precipitation totals, with the elimination of systematic errors of precipitation gauges [5], including wind-induced errors at high winds and false precipitation blown by the wind into the precipitation gauge during strong blizzards at low temperatures were used. Totally data for 141 weather stations in the study area for the period 1979–2015 were collected. Nine weather stations were excluded from the analysis due to more than 5% of missing values. Finally, only reliable data records from 132 ground weather stations were used for bias correction.

*3. Interpolation of reanalysis regular grid data into weather station locations*

The ERA5 monthly total precipitation data from the reanalysis regular grid were interpolated into the weather station coordinates using simple bilinear interpolation for each month and year of the study period [3]:

$$R_{s,m,y}=b_{1}+b_{2}\cdot x_{s}+b_{3}\cdot y_{s}+b_{4}\cdot x_{s}\cdot y_{s},$$

where *x_s_* and *y_s_* are the station (*s*) latitude and longitude, interpolation parameters *b_1_, b_2_, b_3_*, and *b_4_* were calculated from the solving of the system of the linear equations for each station (*s*), month (*m*), and year (*y*) using the ERA5 total precipitation data from four reanalysis grid points surrounding the weather station. The interpolation of reanalysis data to the points of location of weather stations was carried out in order to perform a comparison of these two data sets: reanalysis data and observational data.

After this the mean error (ME), mean absolute error (MAE), mean relative error (MRE), and root mean squared error (RMSE) were calculated for each station and every month to describe the bias of the reanalysis data. Also, three additional statistics were used for characterizing the performance of the reanalysis product: Pearson's linear correlation coefficient (CC), the Nash–Sutcliffe efficiency (NSE [6], and Kling–Gupta efficiency (KGE [7,8]).

Detailed analysis of reanalysis/observations difference is presented in [3].The linear correlation coefficients between reanalysis and weather station data are high but mean values are biased [3]. The mean absolute error varies from −23 to 90 mm.

*4. Calculation of bias correction parameter for weather stations*

For the bias correction of reanalysis data the linear-scaling approach were used that operates monthly correction values based on the differences between observed and reanalysis values. The linear-scaling factors [9] based on the ratio of the long-term total monthly observed and the reanalysis data were calculated for each weather station (*s*) and month (*m*):

$$a_{s,m}=\bar{O_{s,m}}/\bar{R_{s,m}},$$

where $\bar{O_{s,m}}$ and $\bar{R_{s,m}}$  are the long-term mean precipitation for station *s* at month *m* from observations and reanalysis data, $a_{s,m}$ is a scale factor calculated for the whole observation period for each station *s* and month *m*.

*5. Interpolation of bias correction parameter into regular grid*

In order to obtain the scale factor values $a_{i,j,m}$ at each grid point over the study area the ordinary kriging interpolation technique [10] was used. The spatial resolution of the scale factor map corresponds to the ERA5 reanalysis grid (0.25° × 0.25°).

$$a_{i,j,m}=f\left(a_{s,m},\;x_{i},y_{j}\right),$$

*6. Applying bias correction coefficient to ERA5 data subset*

The bias-corrected precipitation $CR_{i,j,m,y}$ at grid points ($x_{i},y_{j}$) over the study area were calculated as the is the monthly precipitation obtained from reanalysis $R_{i,j,s,y}$ scaled using the gridded scale factor $a_{i,j,m}$:

$$CR_{i,j,m,y}=a_{i,j,m}\cdot R_{i,j,m,y},$$

*7. Validation of bias-corrected data*

The performance of the bias-correction procedure was analyzed using the cross-validation method. The full set of data was divided into two parts. The larger subset of samples contains 80% of the data and was used for model construction. The remaining small part of data was used for model verification, called the test set [11]. 106 weather stations were included in the training set, and 27 stations were marked as the test (control) set. To reduce the uncertainties related to the test set selection [12], we generate 200 random nonrepeating training sets (106 stations each) and evaluate the bias correction quality for all 200 test sets (27 stations each). Each weather station was accounted in 200 test sets randomly several times (from 25 to 54). For each station, we calculate averaged statistic characteristics (ME, MAE, MRE, RMSE, CC, NSE, KGE) for the 200 test sets and use them to describe the quality of the bias-corrected data (results see in [3]).

## 3 Conclusions

The bias correction procedure applied to the ERA5 data changes the monthly precipitation sums, resulting in reduced errors. The mean error becomes smaller, and the area-averaged values become almost zero. The linear scaling bias correction method adjusts only the long-term mean values with respect to the observation data and it works well at the monthly scale [13], while the distribution mapping method [14] was able to correct the modelled daily rainfall data more reliably, irrespective of the seasons or climate zones. Nevertheless, even linear correction improves the results of precipitation modelling [15] and changes the monthly precipitation. The precipitation sums after bias correction slightly decrease in the winter months and substantially (by 10–13 mm) decrease in summer. The standard deviation also reduces for all months, but the changes are more pronounced in summer. For the detailed results and analysis (comparison of reanalysis data and in-situ data, bias correction, statistical evaluation of bias correction procedures and discussion), please see the related research article shown below [3].

The obtained results are promising since the proposed simple correction scheme is more accurate and robust in reproducing monthly - precipitation values than global reanalysis data. CPSS reproduces the spatial variability of precipitation more precisely than can be done from the weather station observation network. The CPSS dataset will be useful for the study of extreme precipitation events and allow for more accurate hydrologic risk assessment at a regional level based on climate model results.

## CRediT authorship contribution statement

**Anna Ryazanova:** Software, Validation, Formal analysis. **Nadezhda Voropay:** Writing – review & editing, Conceptualization, Methodology, Investigation, Supervision. **Egor Dyukarev:** Writing – original draft, Methodology, Visualization.

## Declaration of Competing Interest

The authors declare that they have no known competing financial interests or personal relationships which have or could be perceived to have influenced the work reported in this article.
